# Embryonic yield-related serum proteomic analysis at bovine superovulation

**DOI:** 10.3389/fvets.2026.1923597

**Published:** 2026-09-29

**Authors:** Yongli Yue, Anping Xie, Ruilin Fang, Xvhui Hu, Yijing Zhu, Ruifang Li, Liguo Zhang, Xiaohu Su

**Affiliations:** 1 The State Key Laboratory of Reproductive Regulation and Breeding of Grassland Livestock, School of Life Sciences, Inner Mongolia University, Hohhot, China; 2 Ulanqab Animal Husbandry Workstation, Ulanqab Agriculture and Animal Husbandry Bureau, Ulanqab, China; 3 Liangcheng Mai Hutu Town Comprehensive Support and Technology Promotion Center, Ulanqab, China

**Keywords:** superovulation, cattle, serum proteome, biomarker, donor selection

## Abstract

Superovulation is a key technology for accelerating genetic improvement in cattle, but its efficiency is limited by the unpredictable ovarian response among donors. A non-invasive method to predict this response could enhance superovulation outcomes. As serum proteome may mirror ovarian function, this study aimed to identify potential serum protein biomarkers at critical time points to predict embryo yield in donor cows. In this study, blood samples were collected from 33 donor cows undergoing superovulation at two time points: before FSH administration (designated BS) and at estrus detection (designated E). Based on the total number of embryos recovered, the cows were subsequently assigned to high embryonic yield (HEY) and low embryonic yield (LEY) groups. Proteomic sequencing was performed using liquid chromatography-tandem mass spectrometry (LC–MS/MS). A total of 352 embryos were collected. Proteomic analysis identified 1,620 proteins, corresponding to 11,726 annotated entries. A total of 154 and 102 differentially expressed proteins were identified between HEY and LEY at BS and E, respectively (|log_2_FC| ≥0.263, P < 0.05, FDR<0.01). Thirteen proteins were shared between the two time points, including consistently upregulated vitamin D-binding protein (GC). The enriched pathways were mainly metabolism-related. Notably, elevated TCN2 and DNMT1 at BS (AUC = 0.96 and 1.00, respectively), and AGRN, RCN3, and WNT7B at E (AUC = 1.00, 0.80, and 0.84, respectively), were associated with the HEY group, suggesting their potential as biomarkers for predicting embryo yield in bovine superovulation. Collectively, this study identified multiple serum protein biomarkers, providing novel molecular insights into the serum protein response to superovulation and laying a foundation for the development of non-invasive prediction strategies.

## 1 Introduction

Cattle of superior reproductive performance hold substantial economic value in modern livestock production. However, as monotocous animals, cattle exhibit inherently low reproductive efficiency under natural conditions. The application of superovulation technology enables the recovery of multiple fertilizable mature oocytes from elite donors within a single estrous cycle (1). When combined with artificial insemination and embryo transfer, this approach offers a powerful means for rapid genetic dissemination and accelerated breeding progress.

Nevertheless, the ovarian response to superovulation protocols varies considerably among individual donors, leading to unpredictable embryo yields and, in many cases, inefficient use of hormonal preparations and associated costs (2). To improve superovulation efficiency, extensive efforts have been devoted to identifying predictive indicators based on ultrasonographic assessment of ovarian status, genotyping of reproduction-related loci, and analysis of circulating reproductive hormones, vitamins, metabolites, and biochemical parameters (3–11). These studies have generated valuable insights; however, most of the reported associations exhibit poor reproducibility across different breeds. Moreover, the relatively high cost and technical demands of these detection methods have hindered their routine application in breeding programs (12).

The advent of high-throughput “omics” technologies has opened new avenues for deciphering the molecular mechanisms underlying reproductive performance and identifying biomarkers that can predict fertility outcomes in individual animals (13). Recently, transcriptomic analyses of bovine antral follicles have mapped ligand-receptor interactions that predict intrafollicular factors for improving oocyte quality (14), and single-cell RNA sequencing has revealed transcriptome dynamics during bovine oocyte growth from the secondary follicle to the mid-antral stage (15). Metabolomics and lipidomics have also become indispensable tools for elucidating the molecular basis of reproductive performance in livestock (16). A systematic review of oocyte competence in cattle identified nucleotide metabolism and extracellular matrix interactions as key pathways influencing oocyte quality (17).

Proteomic profiling enables comprehensive comparative analysis of all proteins within a given biological sample, thereby facilitating the identification of molecular markers and regulatory pathways associated with specific physiological or pathological phenomena. In livestock studies, proteomic analyses have been increasingly utilized to elucidate the molecular events underlying critical reproductive processes, such as estrous cycling and gestation. For instance, N. G. Shashikumar et al. (11) identified potential stage-specific markers in the saliva of buffaloes across different estrous stages. Ankan De et al. (5) analyzed serum proteomic changes to reveal biomarkers during early pregnancy in pigs. Similarly, Wang et al. employed proteomic analysis in camels to identify potential indicators of reproductive capacity (18). Integrated proteomic profiling of serum, follicular fluid, and ovarian tissues has been used to identify molecular alterations linked to ovarian dysfunction and early pregnancy dynamics in cattle (19, 20). Despite these advances, the correlation between serum protein composition and superovulation yields in cattle has not yet been reported.

To discover serum protein biomarkers associated with superovulation efficiency in cattle, blood samples were collected from Huaxi donor cattle on the day preceding FSH injection and at the time of estrus detection. Based on embryo production, the donors were classified into high-, medium-, and low-yield groups. Comparative serum proteomic analysis was performed between the high- and low-yield groups. The findings of this study are expected to provide a novel theoretical basis for the selection of donor cows in bovine superovulation programs.

## 2 Methods

### 2.1 Experimental location and cows' management

This study was conducted at the Livestock Experimental Base of Inner Mongolia University in Hohhot, Inner Mongolia, China (40°49′N, 111°41′E; elevation 1 050 m). This region has a mid-temperate, semi-arid continental monsoon climate, characterized by long dry winters, short rainy summers, an annual mean temperature of 7.2 °C, and an annual average precipitation of 392.2 mm. A cohort of 33 sexually mature, healthy Huaxi cows, aged 2–4 years and weighing 580 ± 20 kg, with no history of reproductive disorders and no record of two or more unsuccessful breeding attempts, were used as donors in this study. They were raised in free-stall barns and provided with water and shade *ad libitum*. The diet comprised 15.0% alfalfa hay, 50.0% whole-crop corn silage, and 35.0% concentrate supplement (582, Zhengda, China), yielding a forage-to-concentrate ratio of 65:35 (dry matter basis).

### 2.2 Synchronized superovulation and embryo recovery protocol

Cows were synchronized and superovulated according to the schedule shown in Figure 1 (21). On Day 0 (D0), a controlled internal drug-release (CIDR) device containing 1.38 g progesterone (CIDR 1380, Zoetis, New Zealand) was inserted intravaginally. From D9 to D12, a total of 500 μg FSH (Stimufol^®^, Reprobiol, Belgium) was administered intramuscularly (i.m.) in eight step-down doses, each 10 % lower than the previous one. 300 μg prostaglandin F_2_α (010492201, GINTEN, China) was given concurrently with the fifth FSH injection on the morning of D11, and the CIDR was removed at the final FSH injection. Estrus was detected on D13, with the primary criterion being the cow's willingness to stand when mounted. Cows were inseminated twice at 12-h interval. On D20, embryos were recovered by non-surgical uterine flushing under caudal epidural anesthesia with 5 mL of lidocaine hydrochloride (H14024045, CSPC, China).

**Figure 1:**
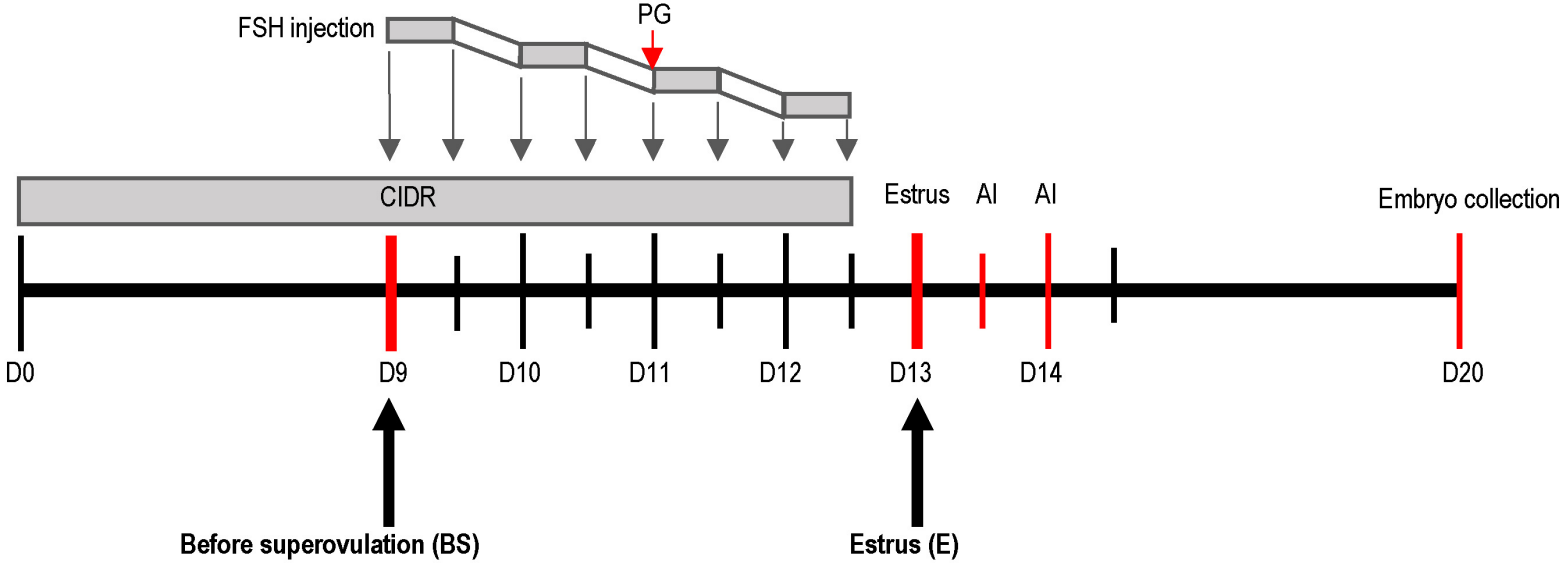
Schematic timeline of the superovulation protocol. A controlled internal drug-release device (CIDR; Zoetis, New Zealand) was inserted intravaginally on D0. From D9 to D12, 500 μg of follicle-stimulating hormone (FSH; Stimufol^®^, Belgium) was administered intramuscularly in eight decreasing doses (10% reduction per dose). On the morning of D11 (the fifth FSH injection), 300 μg of prostaglandin F_2_α (PG; Reprobiol, New Zealand) was given concurrently. The CIDR was withdrawn with the last FSH injection on D12. Estrus was detected on the morning of D13, and artificial insemination (AI) was performed twice at a 12-h interval. On D20, embryos were recovered by uterine flushing. Black arrows indicate the blood sampling time points, designated as BS (before FSH injection) and E (at estrus detection).

### 2.3 Blood sampling

Blood samples were collected into 10-mL serum-separator vacutainers at 8:00 on two time points: the 9th day before FSH administration of superovulation (designated BS time point) and the 13th day of estrus detection (designated E time point). Centrifuge the blood sample at 8,000 × g for 10 min to obtain the serum which was stored in liquid nitrogen for future use. The BS time point corresponds to the pre-stimulation stage when antral follicles are quiescent and have not yet been recruited, whereas the E time point corresponds to the pre-ovulatory stage when follicles have reached maturity.

### 2.4 LC–MS/MS measurements for serum proteomics and data processing

Based on the total embryo yield, donors producing ≥13 viable embryos were designated high-embryo-yield (HEY; *n* = 5), and those producing ≤ 5 embryos were designated low-embryo-yield (LEY; *n* = 5) (22). The Proteomic data analysis was performed by Shanghai Luming biological technology Co., LTD (Shanghai, China). Briefly, the low-abundance proteins of serum samples were enriched with magnetic-bead affinity. The peptides were evaporated and reconstituted in mass spectrometry solvent A (0.1% formic acid in water), followed by quantitative analysis.

TimsTOF HT (Bruker, Germany) and nanoElute2 (Bruker, Germany) were used for both shotgun proteomics and data-independent acquisition (DIA) analyses. Peptide samples were loaded and separated by a C18 column (15 cm x 100 μm, 1.9 μm). The linear gradient was set as follow: 0~7.5 min, 6–28% B; 7.5~9 min, 28–80% B; 9~10min, 80–80% B at 0.7 μL min^−1^. The mass spectrometry parameters were set as follows: the capillary voltage was maintained at 1.6 kV, with primary and secondary scan ranges spanning from 300–1500 m/z, collision energy of 20–59 ev, ramp times of 50 ms and ion mobility windows (1/K0) ranging from 0.7–1.3 Vs/cm^2^.

Default factory settings were used for both database search and spectral library generation in Spectronaut Pulsar 18.7 (Biognosys, Switzerland): trypsin/P was designated as the enzyme, up to two missed cleavages were allowed, methionine oxidation was set as a variable modification, carbamidomethylation as a fixed modification, and a 1% false discovery rate (FDR) was applied at the PSM, peptide, and protein levels. The DIA data were processed with the following parameters: a precursor q-value cutoff of 1%, a protein q-value cutoff of 1%, local normalization as the normalization strategy, and MS2-level quantification. Differentially expressed proteins (DEPs) were identified based on thresholds of fold change >1.2 or < 0.83 and *P*-value < 0.05. Identified proteins were annotated with Gene Ontology (GO, http://geneontology.org/), and Kyoto Encyclopedia of Genes and Genomes (KEGG, http://www.genome.jp/kegg/). Protein-protein interaction (PPI) analysis was performed using the STRING (https://string-db.org/).

## 3 Result

### 3.1 Embryo outcomes

In this study, 33 cows underwent the synchronized superovulation and embryo recovery protocol outlined in Figure 1, and the results are summarized in Table 1. A total of 352 embryos were flushed, yielding an average of 10.67 ± 7.35 embryos per donor, of which 284 were graded as viable, corresponding to a mean of 8.61 ± 6.86 transferable embryos per cow. Animals yielding ≥13 embryos were designated as the high-embryo-yield (HEY) group (*n* = 10), whereas those with ≤ 5 embryos were assigned to the low-embryo-yield (LEY) group (*n* = 8). HEY donors produced 18.70 ± 7.50 total embryos and 16.40 ± 6.36 viable embryos, whereas LEY donors generated 2.88 ± 1.46 total embryos and 2.25 ± 1.16 viable embryos. From each of the HEY and LEY groups, 5 cows were selected for serum proteomic analysis.

**Table 1:** Summary of embryo production following superovulation in cows (mean ± SD).

Donor number	Total embryos	Average total embryos	Total viable embryos	Average viable embryos	Donor number (HEY)	Average total embryos of HEY	Average viable embryos of HEY	Donor number (LEY)	Average total embryos of LEY	Average viable embryos of LEY
33	352	10.67 ± 7.35	284	8.61 ± 6.86	10	18.70 ± 7.50	16.40 ± 6.36	8	2.88 ± 1.46	2.25 ± 1.16

### 3.2 Protein identification and quantitation

The serum proteomic profiling showed that across the 20 proteomic samples (two animal groups, two time points), 1,620 proteins were detected, corresponding to 11,726 peptides. Principal-component analysis (PCA) demonstrated discrete clustering between the HEY and LEY groups. For the BS samples, PC1 and PC2 explained 21.5% and 15.7% of the variance, respectively (cumulative 37.2%, Figure 2A), revealing a clear separation between the two groups. For the E samples, PC1 and PC2 explained 23.6% and 16.4% of the variance (cumulative 40.0%, Figure 2B), but no evident clustering separation was observed. According to the eggNOG Database analysis, the most enriched category was posttranslational modification, protein turnover, chaperones; translation, ribosomal structure and biogenesis; intracellular trafficking, secretion, and vesicular transport; cell wall/membrane/envelope biogenesis (Supplementary Table S1).

**Figure 2:**
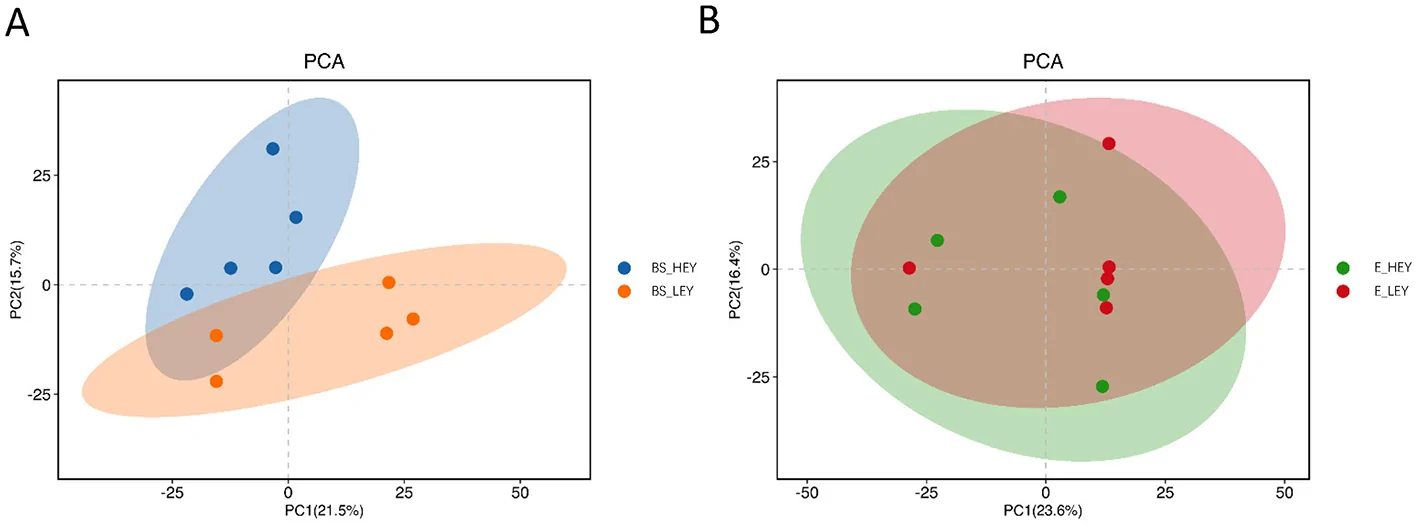
Principal component analysis (PCA) biplot illustrating the separation of protein expression profiles. **(A)** BS-HEY (blue) vs. BS-LEY (yellow). **(B)** E-HEY (green) vs. E-LEY (red).

For the DEPs between the HEY and LEY groups (|log_2_FC| ≥0.263, *P* < 0.05), the number at the BS sampling time point (154) was greater than that at the E time point (102) (Supplementary Table S2). Volcano plot (Figure 3A) showed that, 80 proteins were more abundant and 74 were less abundant in HEY vs. LEY at the BS sampling time point; at the E sampling time point, 58 proteins were up-regulated and 44 down-regulated. Table 2 showed the subset of the top 6 most significantly DEPs among groups. Venn diagram illustrated the overlap of DEPs between the BS and E sampling groups (Figure 3B). 7 proteins were commonly upregulated and 6 proteins downregulated at both sampling time points. Heat map showed that DNAJC8, TOLLIP, vitamin D-binding protein (DBP, also known as GC), KRT13, OLFM4 and LOC618664 were consistently up-regulated, whereas ABCB10, AIDA, CAVIN2 and SCPEP1 were down-regulated across both BS and E sampling time points (Figure 4).

**Figure 3:**
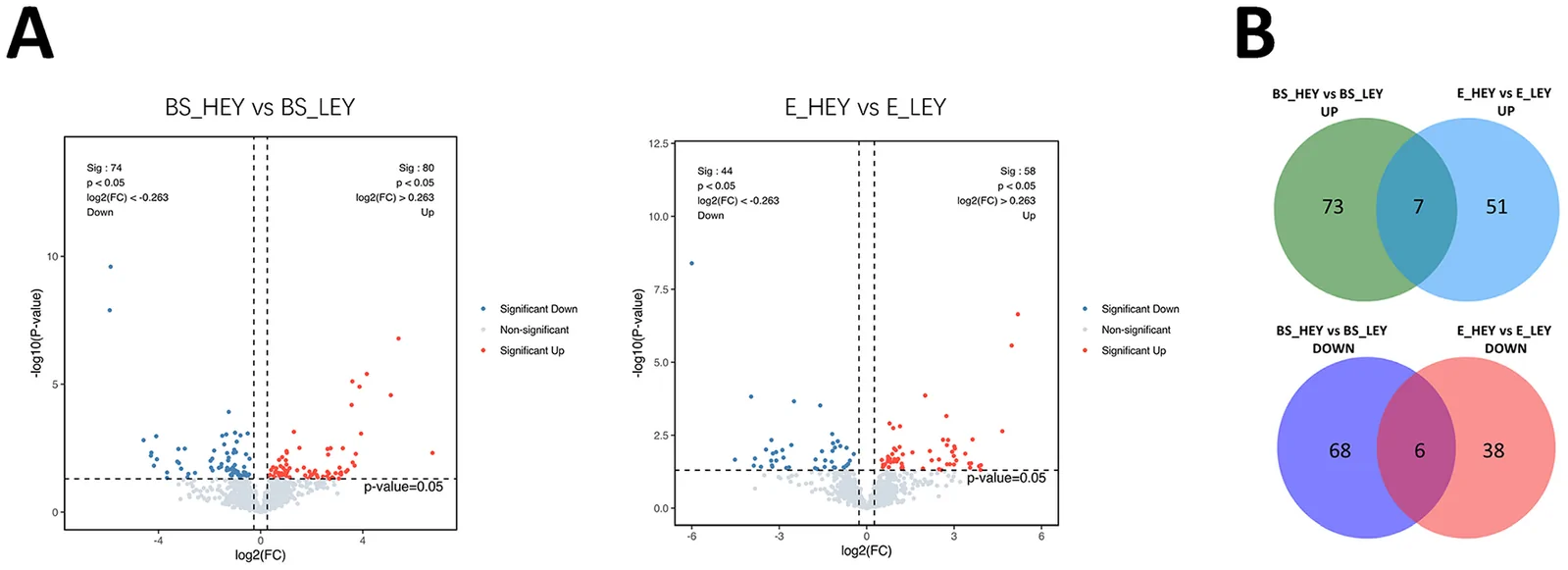
Differential protein analysis results. **(A)** Volcano plot showing the differentially expressed proteins of two sampling time points. **(B)** Venn diagram showing the overlap of up-regulated and down-regulated proteins in the HEY groups and the LEY groups.

**Table 2:** Subset of the top 6 pronounced up- and down-regulated DEPs.

Groups	Gene symbol	Protein name	log2FC	*P*-value
BS-HEY vs. BS-LEY	TCN2	Transcobalamin-2	6.73	< 0.01
	DNMT1	DNA (cytosine-5)-methyltransferase	5.40	< 0.001
	CHL1	Cell adhesion molecule L1 like	5.10	< 0.001
	CDH17	Cadherin 17	4.16	< 0.001
	DAD1	Dolichyl-iphosphooligosaccharide–protein glycosyltransferase subunit	3.93	< 0.001
	DNAJC8	DnaJ heat shock protein family (Hsp40) member C8	3.87	< 0.001
	IGK	IGK protein	−4.18	< 0.05
	NID2	NID2 protein	−4.28	< 0.01
	COX4I1	Cytochrome c oxidase subunit 4 isoform 1, mitochondrial	−4.29	< 0.01
	NA	Ig-like domain-containing protein	−4.58	< 0.01
	KLHL3	Kelch-like protein 3	−5.87	< 0.001
	RAB8B	Ras-related protein 8B	−5.91	< 0.001
E-HEY vs. E-LEY	AGRN	Agrin	5.19	< 0.001
	KRT13	IF rod domain-containing protein	4.98	< 0.001
	IGFN1	Immunoglobulin like and fibronectin type III domain containing 1	4.66	< 0.01
	RCN3	Reticulocalbin 3	3.92	< 0.05
	NPNT	Nephronectin	3.91	< 0.05
	WNT7B	Protein Wnt	3.86	< 0.05
	ARSK	Arylsulfatase K	−3.63	< 0.05
	NA	Ig-like domain-containing protein	−3.83	< 0.05
	NA	Ig heavy chain variable region (fragment)	−3.87	< 0.05
	SCPEP1	Carboxypeptidase	−3.96	< 0.001
	MUC1	Mucin-1	−4.52	< 0.05
	NA	Ig lamda chain variable region (fragment)	−7.70	< 0.001

**Figure 4:**
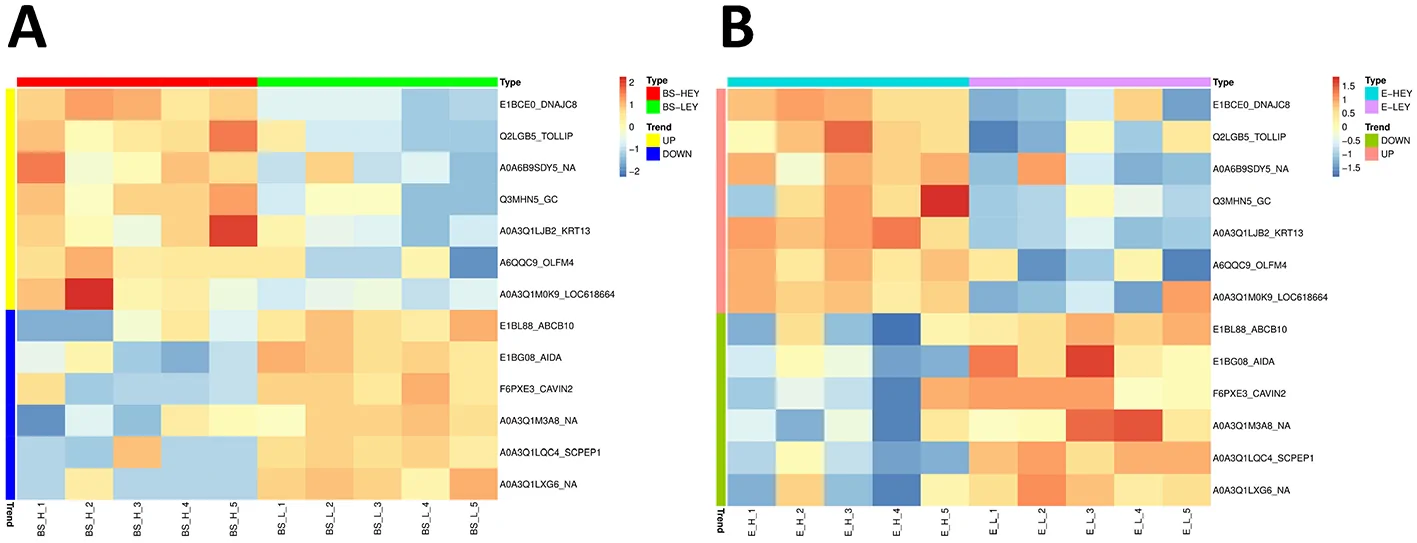
Heat map showing hierarchical clustering of consistently differentially expressed proteins in the HEY and LEY groups. **(A)** BS-HEY vs. BS-LEY. **(B)** E-HEY vs. E-LEY.

### 3.3 Functional analysis of DEPs

Canonical pathway analysis was conducted to determine enrichment of differentially abundant proteins against the background serum proteome. GO analysis of up-regulated DEPs in BS-HEY versus BS-LEY revealed enrichment in biological processes including cellular oxidant detoxification, mRNA splice site recognition, hydrogen peroxide catabolic process, chondrocyte differentiation and extracellular matrix organization; cellular components comprising alveolar lamellar body, haptoglobin-hemoglobin complex, hemoglobin complex, U1 snRNP and extracellular space; and molecular functions encompassing hemoglobin alpha binding, organic acid binding, oxygen carrier activity, oxygen binding and mRNA binding (Supplementary Figure S1A).

For the E-HEY vs. E-LEY comparison, up-regulated DEPs mapped to biological processes of regulation of cell adhesion, glycolysis process, retrograde transport, endosome to Golgi, autophagy and intracellular protein transport; cellular components of basement membrane, endomembrane system, trans-Golgi network, synapse and extracellular region; molecular functions of NAD+-protein-arginine ADP-ribosyitransferase activity, nucleotidyitransferase activity, lipid binding, ubiquitin protein ligase binding and identical protein binding (Supplementary Figure S1B).

KEGG pathway enrichment analysis of up-regulated DEPs revealed comparison-specific signatures. In the BS-HEY vs. BS-LEY comparison, the Classification_level1 terms of DEPs were dominated by Metabolism (particularly glycan biosynthesis, amino acid metabolism, and carbohydrate metabolism), Organismal Systems, Genetic Information Processing, and Environmental Information Processing. Additionally, significant enrichment was found in cobalamin transport and metabolism, vitamin digestion and absorption, cysteine and methionine metabolism, and ECM-receptor interaction (Figure 5A). For the E-HEY vs. E-LEY comparison, the Classification_level1 terms of DEPs were Metabolism (particularly, Amino acid metabolism, Carbohydrate metabolism, and Lipid metabolism), Environmental Information Processing, and Organismal Systems. Notably, steroid biosynthesis and ECM-receptor interaction were significantly enriched (Figure 5B).

**Figure 5:**
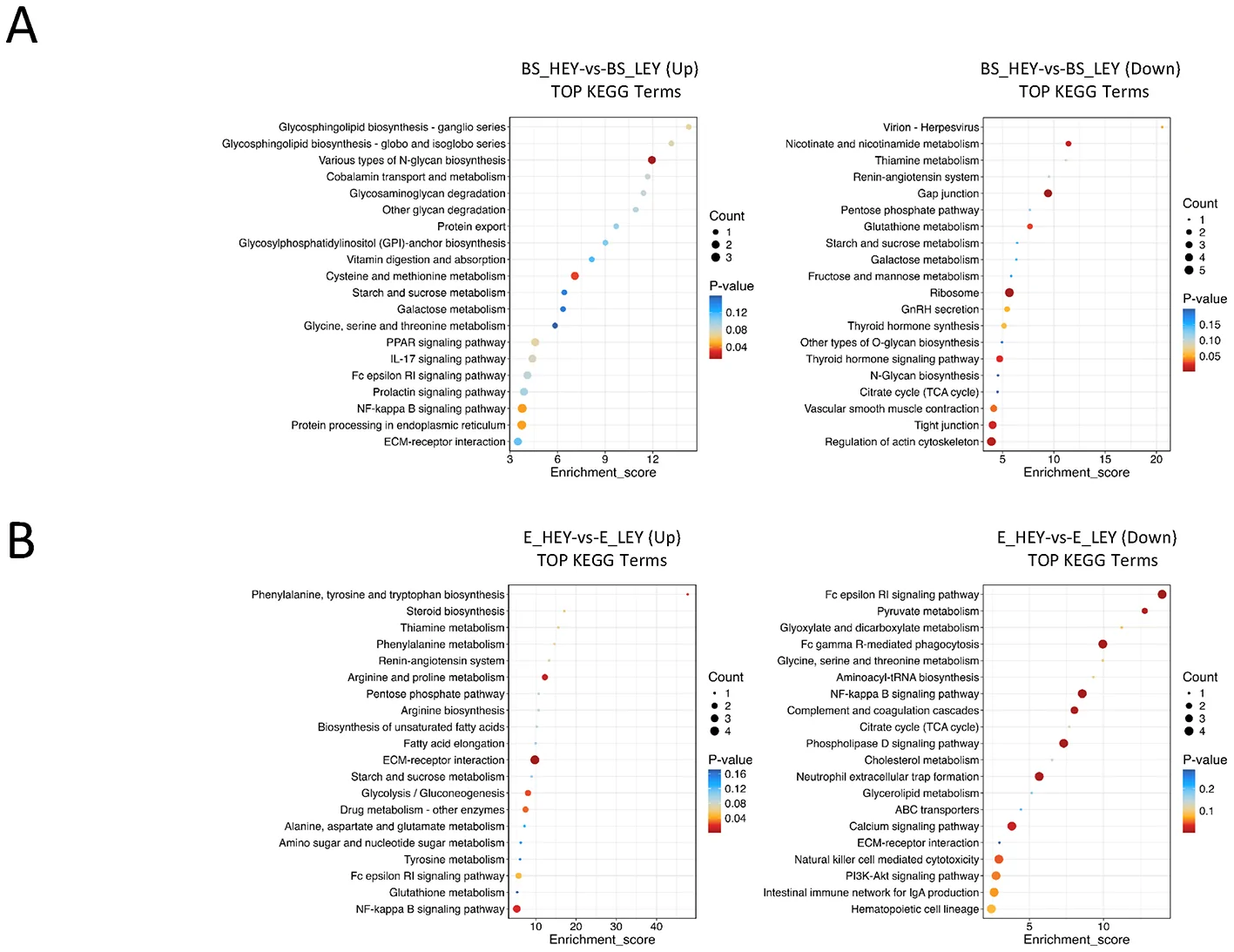
Dot plot showing the top significantly enriched KEGG pathways between groups. **(A)** BS-HEY vs. BS-LEY. **(B)** E-HEY vs. E-LEY.

KEGG-based GSEA identified distinct pathway signatures between the two time points examined (|NES|>1, *P* < 0.05, Q < 0.25) (Supplementary Table S3). The BS time point showed a predominance of negatively enriched pathways (13 with NES < −1 vs. 1 with NES > 1). In contrast, the E time point exhibited a more balanced profile (7 negative vs. 4 positive). Notably, ECM-receptor interaction, cytoskeleton in muscle cells, and human papillomavirus infection pathways exhibited reciprocal regulation, shifting from negative enrichment at the BS time point to positive enrichment at the E time point, suggesting a stage-dependent functional switch during superovulation. The reciprocally regulated pathways collectively encompassed Agrin (AGRN), NPNT, nidogen-2 (NID2), WNT7B, HSPG2, RELN, FN1, ITGA5, FLNC, BGN, DCN, MAP2K1.

### 3.4 Receiver operating characteristic (ROC) curve analysis

To evaluate the predictive performance of the candidate serum proteins for distinguishing HEY donors from LEY donors, receiver operating characteristic (ROC) curve analysis was performed separately for the BS and E time points. The area under the curve (AUC), optimal cut-off value, sensitivity, specificity and Youden index were calculated for each protein (Table 3). At the BS time point, DNMT1 showed perfect discriminative ability (AUC = 1.00, cut-off = 15.43, Youden index = 1.00). GC and TCN2 both exhibited excellent AUC values of 0.96 (cut-offs: 18.23 and 15.14, respectively) and yielded a Youden index of 0.80. For NID2, lower levels could effectively distinguish HEY from LEY donors (AUC = 0.88, cut-off = 10.49, Youden index: 0.80). At the E time point, AGRN exhibited perfect discriminative ability (AUC = 1.00, cut-off = 14.77, Youden index = 1.00), followed by MUC1 (AUC = 0.96, cut-off = 17.49, Youden index: 0.80) and WNT7B (AUC = 0.84, cut-off = 12.98, Youden index: 0.80). However, MUC1 showed an inverse association between protein expression and superovulation outcomes. Both RCN3 and GC showed moderate performance, with AUC values of 0.80 (cut-offs: 14.86 and 16.00, respectively) and Youden index of 0.80.

**Table 3:** Receiver operating characteristic (ROC) curve analysis of candidate protein biomarkers for predicting high embryo yield at the BS and E time points.

Time point	Protein	AUC value	Cut-off value	Sensitivity (%)	Specificity (%)	Youden index
BS	DNMT1	1.00	≥15.43	100	100	1.00
	GC	0.96	≥16.70	100	80	0.80
	TCN2	0.96	≥15.14	100	80	0.80
	NID2	0.88	≤ 10.49	80	100	0.80
E	AGRN	1.00	≥14.77	100	100	1.00
	WNT7B	0.84	≥12.98	100	80	0.80
	GC	0.80	≥16.00	80	100	0.80
	RCN3	0.80	≥14.86	100	80	0.80
	MUC1	0.96	≤ 17.49	100	80	0.80

## 4 Discussion

This study aimed to identify serum protein biomarkers for predicting superovulation efficiency in Huaxi cattle. Serum samples were collected at two critical time points during the superovulation protocol: BS (antral follicle status) and E (mature follicle status). Comparison of the serum proteomic profiles from the same donors at these two time points enables the assessment of dynamic changes in response to superovulation and their association with embryo yield. The proteomic profiling showed that a total of 1,620 proteins were identified from the serum samples of all donor cows, corresponding to 11,726 peptides. PCA revealed a clear clustering separation between the HEY and LEY groups at the BS time point; however, no distinct clustering was observed between the two groups at the E time point. Between HEY and LEY, more DEPs were identified at BS (154) than at E (102), with 80 up-regulated and 74 down-regulated at BS time point, and 58 up-regulated and 44 down-regulated at E time point. Among the DEPs, 7 proteins including GC, were commonly upregulated, while another 6 proteins were consistently downregulated. The DEPs at BS capture the pre-existing molecular differences that may pre-determine the donor's ovarian responsiveness, while those at E capture the molecular events associated with follicular maturation in response to superovulation treatment.

While Amino acid metabolism and Carbohydrate metabolism were enriched at both time points, glycan biosynthesis and metabolism showed a notable BS-specific enrichment, indicating that high-yield donors may possess a more active protein glycosylation. This observation exemplifies the systemic divergence between HEY and LEY groups at the metabolic level. Protein glycosylation, specifically O-GlcNAcylation, acts as a nutrient-sensing mechanism regulating granulosa cell proliferation; its disruption impairs cell expansion and follicular growth in cattle (23). Therefore, the observed pathway enrichment in HEY cows likely reflect an enhanced capacity to sense and utilize energy supply, promoting granulosa cell proliferation and ultimately supporting the recruitment of a larger cohort of follicles and higher embryo yield. By comparison, the E time point featured enrichments in lipid metabolism and steroid biosynthesis. These enrichments align with the role of functional fatty acids in enhancing energy supply and providing hormone precursors to support follicular development (24). For example, Oleic acid, the most abundant free fatty acid in bovine follicular fluid, can directly modulate gonadotrophin receptor expression, steroidogenic enzyme activity, and estradiol-17β synthesis in granulosa cells (25). A proposed regulatory network involving the oocyte-secreted factor FGF10 and Oleic acid appears to modulate the estradiol dominance to progesterone dominance steroidogenic switch in the periovulatory follicle (26). Therefore, the lipid-related pathways enrichment in HEY cows may reflect a more efficient utilization of lipid supply to support the LH-induced steroid shift, thereby promoting oocyte maturation and ovulation.

Among the consistently upregulated proteins in the HEY group, GC is of particular interest, with elevated expression at both time points and enrichment of the vitamin digestion and absorption pathway and high performance of ROC analysis. The sustained GC upregulation in HEY donors may contribute to a favorable peri-ovulatory environment through two potential mechanisms. First, by modulating free 25(OH)D bioavailability, GC may support AMH signaling, FSH sensitivity, and progesterone synthesis (27). Given the strong correlation between serum and follicular fluid 25(OH)D, and its association with improved pregnancy outcomes (28), this mechanism likely facilitates oocyte maturation and implantation. Second, GC may exert vitamin D-independent effects on tissue remodeling and inflammation (29), processes essential for ovulation and luteogenesis. The context-dependent nature of GC function is further supported by genetic evidence: GC enhancer copy number variation in dairy cattle is associated with reduced fertility despite high GC expression (30), whereas the Gc1f genotype in humans confers favorable pregnancy outcomes independent of 25(OH)D levels (31). Thus, while the sustained GC elevation in HEY donors may reflect its multifaceted role in superovulation of Huaxi cattle, the precise contributions of vitamin D-dependent vs. -independent pathways remain to be elucidated through targeted measurement of free 25(OH)D.

At the BS time point, TCN2 was significantly elevated in HEY donors, accompanied by enrichment of the “Cobalamin transport and metabolism” and “Vitamin digestion and absorption” pathways. As the transporter of vitamin B12, TCN2 upregulation suggests enhanced B12 availability in HEY donors. B12 supports oocyte maturation, embryo implantation, and placental development through its roles in folate cycling, one-carbon metabolism, and DNA synthesis (32). The cross-species relevance of this mechanism is supported by evidences from human follicular fluid studies, where B12 levels correlate with ovarian reserve and pregnancy probability (33), and from goat *in vitro* maturation (IVM) models, where B12 supplementation improves fertilization and embryo quality through normalization of DNA methylation (34). Consequently, a GWAS in Gir cattle identified TCN2 as a candidate gene associated with oocyte and embryo counts (35). Furthermore, TCN2 harbors functional variants associated with recurrent implantation failure and elevated estradiol levels in humans (36), further supporting its relevance to reproductive function. While these lines of evidence point to a potential role for TCN2 in superovulation, no functional studies have directly linked TCN2 to reproductive performance in cattle or sheep, warranting further investigation.

DNMT1 was another significantly upregulated protein in the HEY group at the BS sampling time point, a finding supported by the enrichment of the cysteine and methionine metabolism pathway. This pathway generates S-adenosylmethionine (SAM), which serves as the methyl donor for DNMT1 in DNA methylation. DNA methylation plays a central role in gametogenesis, early embryonic development, and epigenetic reprogramming during fertilization. In young lamb donors, insufficient DNMT1 abundance leads to reduced global DNA methylation levels during gametogenesis and early embryonic development, underlying the compromised developmental competence of oocytes and resultant early embryos (37). Studies in yak have also demonstrated that DNMT1 expression in mature cumulus–oocyte complexes play an important role in optimizing sperm–oocyte interactions and DNA methylation patterns during fertilization (38). Notably, the synchronous elevation of DNMT1 and TCN2 (which provides methyl donors) at the BS time point suggests a pre-established cascade in HEY donors, from enhanced vitamin B12 transport to active one-carbon metabolism, sufficient DNMT1 activity, and consequently improved granulosa cell proliferation and epigenetic remodeling, which may underpin their superior superovulation response. ROC analysis also highlights the potential of DNMT1 and TCN2 as non-invasive biomarkers for predicting superovulation outcomes in cattle.

NID2 was highly expressed in the LEY group but significantly downregulated in the HEY group at BS, suggesting that NID2 may serve as a negative selection factor. NID2 is a key component of the extracellular matrix (ECM) and participates in the construction and remodeling of the follicular basement membrane (39). In healthy bovine follicles, NID2 is predominantly localized to the basement membrane, but is aberrantly overexpressed in the granulosa cell layer in atretic follicles (40). The high NID2 expression in LEY donors aligns with this atretic phenotype, whereas its downregulation in HEY donors points to a healthier follicular status, potentially reflecting a more favorable response to subsequent FSH stimulation.

At the E time point, AGRN was significantly elevated in the HEY group and enriched in the ECM-receptor interaction pathway, which has the perfect performance of ROC analysis. AGRN is a multifunctional extracellular matrix protein that exerts its core functions as a heparan sulfate proteoglycan (HSPG). Although its classic role is in the formation and maintenance of the neuromuscular junction, recent studies have revealed multiple reproductive functions of AGRN. AGRN was found enhancing the proliferation of mouse female germline stem cells (41). AGRN also plays an important role in mammalian embryo implantation by its translocating to the apical membrane of the uterine luminal epithelium with co-protein AChR, suggesting that a neuronal signaling system is co-opted in the uterus to direct epithelial closure after implantation (42). The elevated AGRN expression in the E-HEY group together with the perfect performance of ROC analysis, may indirectly suggest a role in uterine or ovarian tissue remodeling, but this interpretation requires functional validation.

RCN3 is another protein elevated in the HEY group at the E time point. As a calcium-binding molecular chaperone localized in the endoplasmic reticulum, RCN3 participates in protein biosynthesis and transport (43). During superovulation, FSH stimulation recruits a large cohort of small follicles into the growth trajectory, a process that partially recapitulates the activation of the primordial follicle pool during fetal development. Our observation of elevated RCN3 in the E-HEY group is consistent with transcriptomic evidence showing higher RCN3 expression in granulosa cells of large, healthy bovine follicles (44). The association between RCN3 and a healthy follicular status is further supported by proteomic analysis of porcine fetal ovaries revealing high RCN3 expression during primordial follicle formation and its contribution to the establishment of the primordial follicle pool (45). Moreover, RCN3 was identified as a candidate gene associated with early pregnancy in Nelore heifers by genome-wide association study (GWAS) analysis (46). Collectively, our serum proteomic data and previous findings reinforce the association of RCN3 with follicle health and dominance throughout the entire life cycle of follicular development, suggesting its potential as a marker of follicle development and recruitment efficacy.

At the E time point, WNT7B was significantly upregulated in the HEY group, while GSK3A (a core kinase of the β-catenin degradation complex) showed downregulated expression and appeared in the PPI network (Supplementary Figure S2). The co-occurrence of WNT7B upregulation and GSK3A downregulation suggests activation of Wnt pathway in the HEY group. This observation aligns with the known role of canonical WNT signaling in supporting oocyte growth during primordial follicle activation, as pre-granulosa cells express multiple WNT ligands that act in an autocrine manner to activate themselves (47). Moreover, granulosa cells of dominant follicles in buffalo show markedly higher WNT7B mRNA expression and the nucleocytoplasmic distribution of β-catenin than those in regressing follicles, consistent with functional evidence from granulosa-oocyte co-cultures (48). WNT7B not only functions in the ovary but also plays a role in the cyclic changes of the uterus, as WNT7B is upregulated by estrogen in the mouse endometrial epithelium and participates in embryo implantation (49). Therefore, the elevated WNT7B at the E time point may reflect more active follicular recruitment and development in HEY individuals in response to superovulation stimulation.

At the E time point, Mucin 1(MUC1) expression was significantly decreased in the HEY group and simultaneously emerged as a central hub in PPI network and exhibited excellent discriminative ability by ROC. In bovine endometrium, MUC1 localizes to the apical surface of the luminal epithelium, with its expression regressing during the luteal phase and gestation, indicating that MUC1 downregulation is a prerequisite for uterine receptivity, and its expression is inversely correlated with fertility (50). In repeat breeder cows, persistent or aberrant MUC1 overexpression disrupts endometrial receptivity and impedes embryo implantation, further supporting the importance of MUC1 downregulation for successful implantation (51). Therefore, the lower MUC1 levels in the E-HEY group may reflect a potentially more implantation-permissive uterine environment, supporting the overall reproductive advantage of these high-yield donors.

Despite these promising findings, the present study was limited to a relatively small sample size (*n* = 33) in Huaxi cattle. Therefore, these findings should first be validated in larger populations across broader age ranges and different superovulation protocols, and ultimately in other cattle breeds. The correlative nature of proteomic data necessitates functional validation in granulosa cell or oocyte models to establish causality. The mechanistic network linking the candidate proteins to ovarian responsiveness, particularly their interconnections and interactions with known reproductive pathways, remains largely undefined. Future studies incorporating larger cohorts, multi-center designs, and functional experiments will be essential to translate these findings into a practical, non-invasive biomarker panel for donor selection in bovine superovulation programs.

## 5 Conclusion

Our findings reveal distinct serum proteomic profiles between high- and low-embryo-yield donors, particularly in metabolism-related pathways. GC emerged as a consistent biomarker, with elevated expression at both time points in the HEY group. Moreover, high levels of TCN2 and DNMT1 at BS, and AGRN, RCN3, and WNT7B at E, were indicative of superior embryo yield, while low NID2 at BS and low MUC1 at E were similarly associated with favorable outcomes. ROC analysis supported the discriminative potential of these proteins as candidate biomarkers. In summary, GC along with other candidate proteins demonstrate potential as serum biomarkers for predicting superovulation outcomes, though their utility in larger populations and production settings awaits further validation.

## Data Availability

The datasets presented in this study can be found in online repositories. The names of the repository/repositories and accession number(s) can be found in the article/Supplementary material.

## Supplementary material

Supplementary Table S1 Statistical summary of eggNOG functional categories.

Supplementary Table S2 List of significantly differentially expressed proteins in the comparisons of BS-HEY vs. BS-LEY and E-HEY vs. E-LEY.

Supplementary Table S3 List of distinct pathway signatures identified by KEGG-based GSEA between the two examined stages.

Supplementary Figure S1 Dot plot showing the top significantly enriched GO pathways among differentially expressed proteins. (A). BS-HEY vs. BS-LEY. (B). E-HEY vs. E-LEY.

Supplementary Figure S2 PPI network of differentially expressed proteins between E-HEY vs. E-LEY.
