# Supplementary material for: Embryonic yield-related serum proteomic analysis at bovine superovulation
Source: Front Vet Sci. 2026 Sep 29;13:1923597. doi: 10.3389/fvets.2026.1923597 (PMC13660016; doi:10.3389/fvets.2026.1923597)
Supplement: Supplementary file 1 [file Image_1.pdf]

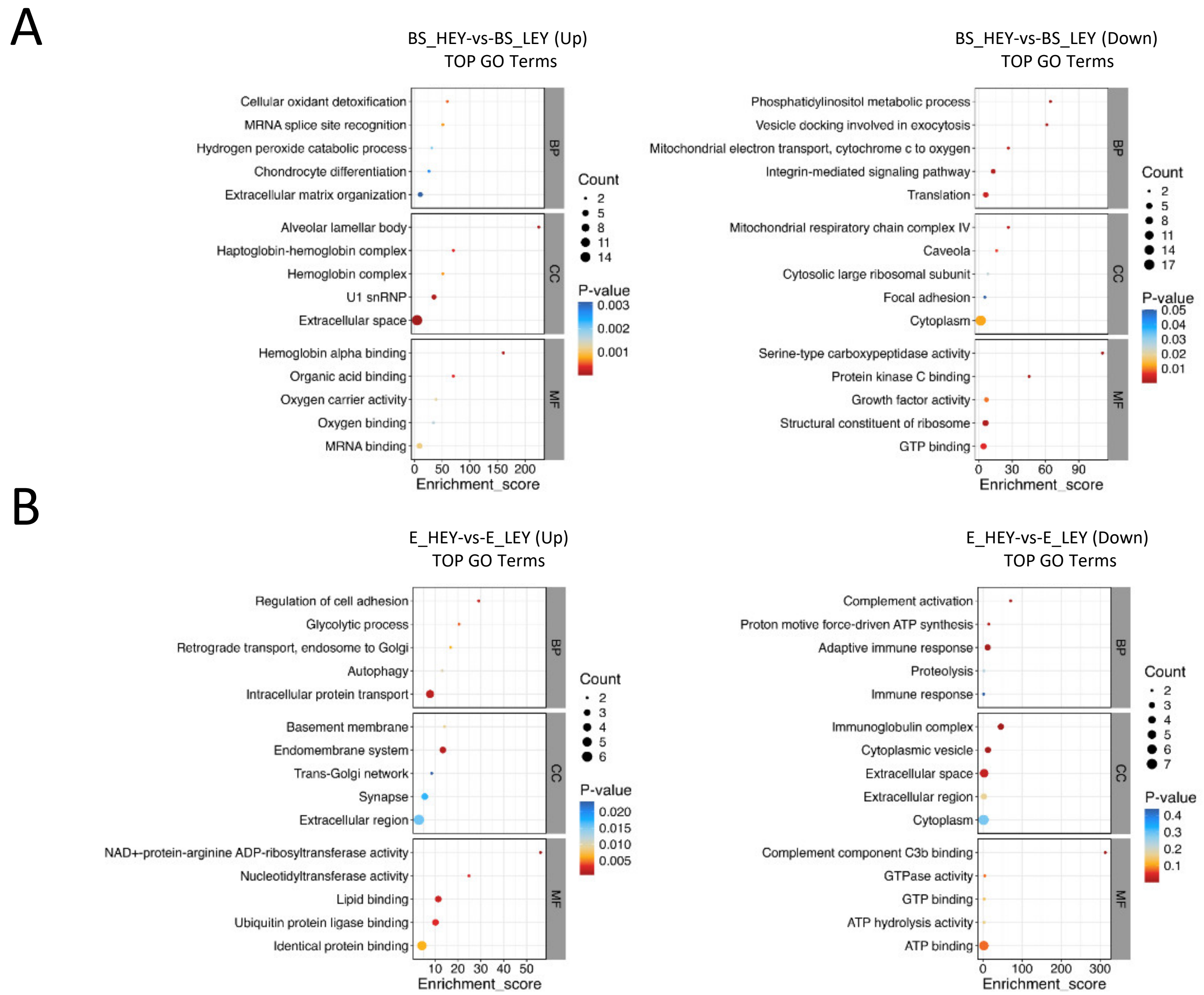
A
BS_HEY-vs-BS_LEY (Up)
TOP GO Terms
Cellular oxidant detoxification
MRNA splice site recognition
Hydrogen peroxide catabolic process
Chondrocyte differentiation
Extracellular matrix organization
Alveolar lamellar body
Haptoglobin-hemoglobin complex
Hemoglobin complex
U1 snRNP
Extracellular space
Hemoglobin alpha binding
Organic acid binding
Oxygen carrier activity
Oxygen binding
MRNA binding
BP
CC
MF
0 50 100 150 200
Enrichment_score
Count
2
5
8
11
14
P-value
0.003
0.002
0.001
BS_HEY-vs-BS_LEY (Down)
TOP GO Terms
Phosphatidylinositol metabolic process
Vesicle docking involved in exocytosis
Mitochondrial electron transport, cytochrome c to oxygen
Integrin-mediated signaling pathway
Translation
Mitochondrial respiratory chain complex IV
Caveola
Cytosolic large ribosomal subunit
Focal adhesion
Cytoplasm
Serine-type carboxypeptidase activity
Protein kinase C binding
Growth factor activity
Structural constituent of ribosome
GTP binding
BP
CC
MF
0 30 60 90
Enrichment_score
Count
2
5
8
11
14
17
P-value
0.05
0.04
0.03
0.02
0.01
B
E_HEY-vs-E_LEY (Up)
TOP GO Terms
Regulation of cell adhesion
Glycolytic process
Retrograde transport, endosome to Golgi
Autophagy
Intracellular protein transport
Basement membrane
Endomembrane system
Trans-Golgi network
Synapse
Extracellular region
NAD+-protein-arginine ADP-ribosyltransferase activity
Nucleotidyltransferase activity
Lipid binding
Ubiquitin protein ligase binding
Identical protein binding
BP
CC
MF
10 20 30 40 50
Enrichment_score
Count
2
3
4
5
6
P-value
0.020
0.015
0.010
0.005
E_HEY-vs-E_LEY (Down)
TOP GO Terms
Complement activation
Proton motive force-driven ATP synthesis
Adaptive immune response
Proteolysis
Immune response
Immunoglobulin complex
Cytoplasmic vesicle
Extracellular space
Extracellular region
Cytoplasm
Complement component C3b binding
GTPase activity
GTP binding
ATP hydrolysis activity
ATP binding
BP
CC
MF
0 100 200 300
Enrichment_score
Count
2
3
4
5
6
7
P-value
0.4
0.3
0.2
0.1
