# Supplementary material for: Embryonic yield-related serum proteomic analysis at bovine superovulation
Source: Front Vet Sci. 2026 Sep 29;13:1923597. doi: 10.3389/fvets.2026.1923597 (PMC13660016; doi:10.3389/fvets.2026.1923597)
Supplement: Supplementary file 2 [file Image_2.pdf]

E_HEY vs E_LEY

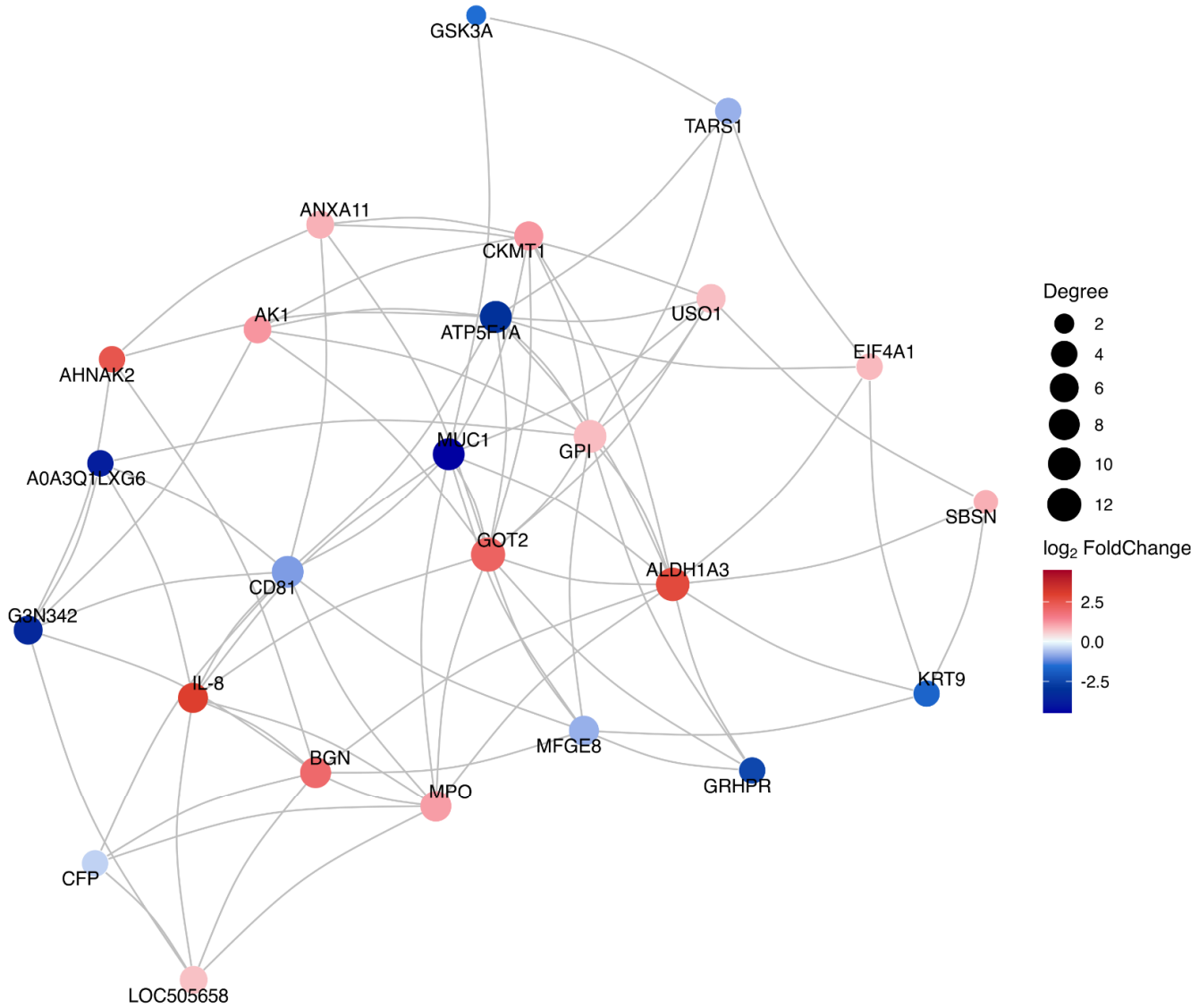

GSK3A
TARS1
ANXA11
CKMT1
AK1
ATP5F1A
USO1
AHNAK2
EIF4A1
A0A3Q1LXG6
MUC1
GPI
SBSN
GOT2
CD81
ALDH1A3
G3N342
IL-8
KRT9
BGN
MFGE8
GRHPR
MPO
CFP
LOC505658
Degree
2
4
6
8
10
12
log2 FoldChange
2.5
0.0
-2.5

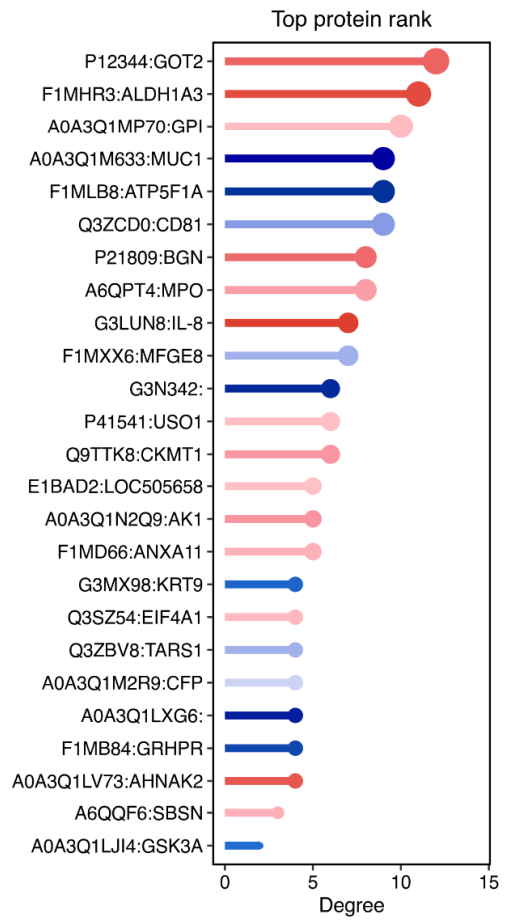

Top protein rank
P12344:GOT2
F1MHR3:ALDH1A3
A0A3Q1MP70:GPI
A0A3Q1M633:MUC1
F1MLB8:ATP5F1A
Q3ZCD0:CD81
P21809:BGN
A6QPT4:MPO
G3LUN8:IL-8
F1MXX6:MFGE8
G3N342:
P41541:USO1
Q9TTK8:CKMT1
E1BAD2:LOC505658
A0A3Q1N2Q9:AK1
F1MD66:ANXA11
G3MX98:KRT9
Q3SZ54:EIF4A1
Q3ZBV8:TARS1
A0A3Q1M2R9:CFP
A0A3Q1LXG6:
F1MB84:GRHPR
A0A3Q1LV73:AHNAK2
A6QQF6:SBSN
A0A3Q1LJI4:GSK3A
0
5
10
15
Degree
